# Microbiota in neuroinflammation and synaptic dysfunction: a focus on Alzheimer’s disease

**DOI:** 10.1186/s13024-022-00522-2

**Published:** 2022-03-05

**Authors:** Diane Bairamian, Sha Sha, Nathalie Rolhion, Harry Sokol, Guillaume Dorothée, Cynthia A. Lemere, Slavica Krantic

**Affiliations:** 1 Sorbonne Université, Inserm, Centre de Recherche Saint-Antoine, CRSA, Immune System and Neuroinflammation Laboratory, Hôpital Saint-Antoine, F-75012 Paris, France; 2 Department of Physiology, Nanjing Medical University, Nanjing, 211166 China; 3 Sorbonne Université, Inserm, Centre de Recherche Saint-Antoine, CRSA, Microbiota, Gut and Inflammation Laboratory, Hôpital Saint-Antoine, F-75012 Paris, France; 4 Paris Center for Microbiome Medicine, PaCeMM, FHU, Paris, France; 5 Gastroenterology Department, AP-HP, Saint Antoine Hospital, F-75012 Paris, France; 6 INRAE Micalis & AgroParisTech, Jouy en Josas, France; 7 Brigham and Women’s Hospital, Harvard Medical School, Boston, MA02115 USA

**Keywords:** Gut microbiota, Synaptic dysfunction, Alzheimer’s disease, Peripheral immunomodulation, Neuroinflammation

## Abstract

**Background:**

The implication of gut microbiota in the control of brain functions in health and disease is a novel, currently emerging concept. Accumulating data suggest that the gut microbiota exert its action at least in part by modulating neuroinflammation. Given the link between neuroinflammatory changes and neuronal activity, it is plausible that gut microbiota may affect neuronal functions indirectly by impacting microglia, a key player in neuroinflammation. Indeed, increasing evidence suggests that interplay between microglia and synaptic dysfunction may involve microbiota, among other factors. In addition to these indirect microglia-dependent actions of microbiota on neuronal activity, it has been recently recognized that microbiota could also affect neuronal activity directly by stimulation of the vagus nerve.

**Main messages:**

The putative mechanisms of the indirect and direct impact of microbiota on neuronal activity are discussed by focusing on Alzheimer’s disease, one of the most studied neurodegenerative disorders and the prime cause of dementia worldwide. More specifically, the mechanisms of microbiota-mediated microglial alterations are discussed in the context of the peripheral and central inflammation cross-talk. Next, we highlight the role of microbiota in the regulation of humoral mediators of peripheral immunity and their impact on vagus nerve stimulation. Finally, we address whether and how microbiota perturbations could affect synaptic neurotransmission and downstream cognitive dysfunction.

**Conclusions:**

There is strong increasing evidence supporting a role for the gut microbiome in the pathogenesis of Alzheimer’s disease, including effects on synaptic dysfunction and neuroinflammation, which contribute to cognitive decline. Putative early intervention strategies based on microbiota modulation appear therapeutically promising for Alzheimer’s disease but still require further investigation.

## Background

Neurodegenerative diseases, such as Alzheimer’s, Parkinson’s and Huntington’s diseases, are age-related neurodegenerative disorders, diagnosed clinically years after the pathogenesis has begun [1]. The development of a chronic inflammatory response in the brain, known as neuroinflammation, is a common early pathological alteration in these disorders [2]. Remarkably, synaptic dysfunction also occurs early in these pathologies, including during the pre-symptomatic stage [3]. However, the link between neuroinflammation and synaptic dysfunctions, used as a proxy of cognitive impairments during the pre-symptomatic stage of these pathologies when cognitive symptoms are still minor or undetectable, is not well understood.

The gut microbiota has recently emerged as an important contributor to Central Nervous System (CNS) homeostasis and dysfunction [4, 5]. Gut microbiota alterations, in addition to the well-established association with gastrointestinal disorders, may increase both intestinal and blood–brain-barrier (BBB) permeabilities. These altered permeabilities may contribute to promoting brain accumulation of gut microbiota-derived molecules (e.g. lipopolysaccharides) and metabolites (e.g. Short Chain Fatty Acids, SCFA) with a subsequent alteration of the homeostatic towards pro-inflammatory conditions and thus set the frame for the pathogenesis of neurodegenerative disorders like Parkinson's disease (PD), Alzheimer's disease (AD), multiple sclerosis (MS) and amyotrophic lateral sclerosis (ALS) [6]. Additional pathological alterations triggered by microbiota could rely on an increase in circulating levels of their metabolites, as well as in humoral (e.g. pro-inflammatory cytokines) or cellular (e.g. monocytes) effectors of peripheral immunity. Along this line, microbiota modulate the regulatory T-cells (Treg) induction [7] as well as the function of microglia, which correspond functionally to brain-resident macrophages [8]. Altogether, this cross-talk between the peripheral immune system and central neuroinflammatory response may be a part of neuropathogenesis in some neurodegenerative disorders [9–11].

Based on this recent knowledge, a new concept has emerged during the last few years, suggesting that microbiota may play an instrumental role in the interactions between peripheral immune response, neuroinflammation and neurodegeneration. However, the precise underlying mechanisms are still to be uncovered. In this review, we first present the data pointing to the interplay between neuronal activity, immune system mediators and microbiota in a broad physiological *versus* pathological context. The emerging role of microbiota is then discussed in the particular context of AD. We next provide a comprehensive multidisciplinary overview, across multiple fields (neuroscience, immunology, microbiology) meant to facilitate the reading for a broad readership from different fields, and further discuss available data on the impact of microbiota on synaptic function in the context of neuroinflammation. In the last part, we address the possible strategies based on microbiota modulation for improving AD-related neuronal dysfunction, either indirectly (via peripheral modulation of inflammatory tone by targeting the microbiota) or directly (via vagus nerve stimulation). We finally discuss the putative therapeutic value of microbiota modulation for early interventions in AD and the perspectives of its translation to other neurodegenerative pathologies.

## Immunomodulatory actions of microbiota

Gut microbiota refers to the microorganisms comprising bacteria, archae, viruses, protists and fungi [6] that colonize the intestine of vertebrates and at least some non-vertebrates such as insects. The diversity and abundance of gut microbiota are host-specific and determined by many factors, including genetic, nutritional and environmental cues. The capacity of microbiota to modulate both peripheral and central immune responses is increasingly recognized. According to an emerging point of view, in addition to its direct impact on neuroinflammation, the gut microbiota can impact the brain immune homeostasis also via modulating peripheral immunity. In this section, we will first discuss the impact of microbiota on the peripheral immune response, then assess the relationship between such microbiota-mediated peripheral immune modulation and neuroinflammation. Finally, we also present recent evidence on the direct effect of microbiota on brain-resident immune responses.

### Impact of microbiota on peripheral inflammation / immunity

Inflammation is triggered by pathogenic microbial molecules known as PAMPs/MAMPs (pathogen-associated molecular patterns / microbe-associated molecular patterns), or by endogenous molecules released by host cells (tumor cells, dead or dying cells, etc.) such as DAMPs (damage-associated molecular patterns). These stereotyped molecular patterns are recognized by tissue-resident immune cells (e.g. macrophages and mast cells), via host PRRs (Pattern Recognition Receptors), which elicit an innate immune response resulting in increased production of cytokines and chemokines and may also include complement activation [12]. Resident macrophages and dendritic cells act as antigen-presenting cells (APC). Upon activation, these APCs migrate to tissue-draining lymph nodes where they present foreign antigens to local immune cells via molecules of the major histocompatibility complex (MHC) to trigger an adaptive immune response. A non-resolved inflammatory response yields recruitment of circulating leukocytes, including effectors of cellular adaptive immunity, i.e. T-lymphocytes, which infiltrate the tissue [12].

Gut microbiota plays multiple roles in humans by constantly interacting with the host immune system through the activation of PRRs expressed by innate and adaptive immune effectors. For instance, lipopolysaccharide (LPS) derivative from the wall of Gram-negative bacteria interacts with TLR4 (Toll-like receptor-4) [13], a PRR expressed not only by innate and adaptive immune cells but also by intestinal epithelial cells. Alteration in gut microbiota composition and function (dysbiosis) may play a role in overactivating the intestinal immune system, inducing gut barrier dysfunction [14].

Additional routes of microbiota-immune system communication rely on the production of a variety of signaling molecules by the gut microbiota. Such immuno-active signaling molecules include notably: (i) SCFA, generated by bacteria from the fermentation of undigested fibers; (ii) secondary bile acids issued by microbiota transformation of primary bile acids produced in the liver, and (iii) tryptophan metabolites [15, 16]. Metabolites production depends therefore on host diet and microbiota composition. SCFAs contribute to immune homeostasis in mucosal and systemic compartments. For example, SCFAs produced by *Clostridia* are involved in the activation and expansion of Tregs [17, 18]. Other SCFAs, such as propionate, acts directly on γδ T-cell subpopulation to inhibit their production of interleukin-17 (IL-17) in both mice and humans (i.e. patients with inflammatory bowel disease) [19]. Bile acids trigger the activation of farnesoid-X-receptor (FXR) and the G protein-coupled bile acid receptor 1 (GPBAR1), which are highly expressed in innate immune cells, including intestinal macrophages, dendritic cells, and natural killer T-cells, and contribute to maintaining intestine immune functions [20]. Tryptophan is an essential amino acid, but it also serves as a precursor for a large number of bioactive compounds such as indoles, tryptamine, serotonin and kynurenine [4, 21]. Indole derivatives, which are produced exclusively by the gut microbiota, impact the functional differentiation of naive CD4 + T cells into Tregs and T-helper 17 (Th17) cells [21]. Indoles are also involved in the control of mucosal Tregs/Th17 ratio and thus anti-/pro-inflammatory balance in different compartments of the gastrointestinal tract [22]. Of note, gut-activated Th17 cells trigger T cell-dependent high-affinity Immunoglobulin A (IgA) secretion [23].

However, the interplay between microbiota and immune cells may be more complex and include additional, previously uncovered players such as enteric neurons. A very recent study reported a regulatory circuit wherein microbial signals, likely (but not exclusively) LPS or pore-forming toxins, condition enteric neuron activation and associated neuronal interleukin-6 (IL-6) induction, which subsequently tunes gut RORγ + subset of Tregs to control immunological tolerance [24]*.*

To sum up, the gut microbiota influences the cellular function and migration properties of various immune cells subsets, including peripheral myeloid cells, T cells and mast cells [7]. In addition to regulating the systemic immune responses, intestinal bacteria also influence mucosal immunity that plays a role in host defense against pathogens [5, 7].

### Impact of microbiota on neuroinflammation

#### Indirect actions via peripheral and central immune cross-talk

Microbiota can also influence inflammation in the brain (neuroinflammation) via interactions between the CNS and the gut along the “microbiota-gut-brain axis”. This axis refers to a complex network of interactions allowing for bidirectional communication between the gut microbiota and the CNS. The relevant interactions involve both cellular (e.g. via immune cells…) and humoral (e.g. via cytokines…) modes of communication.

Maintenance of CNS homeostasis involves immune surveillance by patrolling T cells and mature APCs that are confined to perivascular spaces, meningeal areas and the choroid plexus. In particular, activated myeloid and lymphoid immune cells present in the brain meninges produce cytokines that are drained to the cerebro-spinal fluid (CSF) and can be transported via the glymphatics system to the brain parenchyma, where they modulate glial cells (microglia, astrocytes and oligodendrocytes). These latter are the major source of endogenous cytokine production in the brain. Both brain- and peripheral-derived cytokines can also impact the BBB permeability, for instance by reducing the expression of the tight junction proteins between endothelial cells [25]. Besides, in pathological settings, activated immune cells can enter into the CNS parenchyma through the superficial leptomeningeal vessels and choroid plexus [26, 27], and this process is further amplified upon permeabilization of BBB [25]. Moreover, pro-inflammatory cytokines produced by competent brain glia cells increase the expression of adhesion molecules such as selectins and integrins on cerebral endothelial cells and thus facilitate T cells infiltration into the brain [27]. In the context of systemic inflammatory conditions, the impact of increased peripheral cytokine production may therefore be further enhanced by endogenous cytokine induction in the brain, thus setting the frame for a feed-forward escalation of brain inflammatory response [25].

Among all glia cells, microglia are the major source of cytokines in the CNS. These brain-resident immune cells are able to drive innate immune responses and act as APCs. Depending on environmental cues, microglia display a variety of phenotypes [28–31]. From a functional point of view, microglia can adopt a wide range of reactivity states, going from homeostatic, which promotes neuronal health and survey the CNS microenvironment, to pathologically activated states that are characterized by various patterns of cytokine production and/or phagocytosis, sometimes turning excessive [29, 30]. These functional states of microglia span a large spectrum of morphologies ranging from a ramified “homeostatic” morphology at steady state, to diverse “polarized” morphologies with contracted processes and cell bodies (“amoeboid state”) in pathological conditions and during aging, including a dystrophic morphology associated with chronic neuroinflammation and neurodegeneration. Recent wide-scale single-cell transcriptomic analyses allowed for a more precise molecular characterization of microglia. These studies established a specific transcriptomic signature, which differs between homeostatic (M0) and Disease-Associated Microglia (DAM), the latter being similar to MicroGlia in neuroDegeneration [MGnD] profile [28–30]. Although the previous distinction between M1 (classical, pro-inflammatory) and M2 (alternative, anti-inflammatory) polarized states of microglia [31] is now considered as an oversimplified classification, it could still be helpful for illustrating two extreme functional profiles of microglia/macrophage diversity. The previously named M1-like phenotype of microglia, by analogy to macrophages, can be promoted by cytokines produced by CNS-patrolling or infiltrated T-helper 1 (Th1) or Th17 CD4 + T-cells. Such pro-inflammatory Th1-derived (e.g. Interferon gamma [IFNγ], Tumour necrosis factor alpha [TNFα]) and/or Th17-derived (e.g. GM-CSF) cytokines foster the microglia/macrophages response to PAMP/DAMP, resulting in increased production of pro-inflammatory cytokines (TNFα, interleukin-1 beta [IL-1β], IL-6, interleukin-12 [IL-12], etc.…) as well as an increase in reactive oxygen species (ROS) and nitrogen reactive species (NOS) [31]. On the other hand, the M2-like phenotype is promoted by T-helper 2 (Th2)-derived (e.g. interleukin-4 [IL-4], interleukin-5 [IL-5], interleukin-13 [IL-13]) and/or Treg-derived (e.g. interleukin-10 [IL-10], Transforming growth factor beta [TGFβ]) cytokines, resulting in activated microglia/macrophages that subsequently respond by increased production of anti-inflammatory cytokines and neurotrophic factors, as well as enhanced phagocytosis [32, 33].

During recent years, a new concept emerged pointing to a putative role of microbiota in the above mentioned cross-talk between peripheral and central inflammation. For instance, peripheral injection of LPS as well as heat-killed or live pathogens induces an immunological response in the brain of rodents by promoting microglia activation, as reported in a recent comprehensive review [34]. Such microglia activation is associated with neuroinflammation, including up-regulation of TLR2, TLR4, TNFα and IL-1β at both ribonucleic acid (RNA) and protein levels [34]. However, the latter systematic review covered the studies published prior to the description of transcriptional M0 vs. DAM/MGnD profiles, and there is so far no equivalent overview based on the analysis of transcriptomic phenotypes. Of note, recent studies reported that different regimens of peripheral LPS injection -associated with different blood levels of pro-inflammatory cytokines- had opposing effects on microglia and disease progression in a mouse model of amyloid pathology [35]. Treatment regimens associated with enhanced peripheral pro-inflammatory profiles resulted in microglia displaying an activated profile, which exacerbates neuroinflammatory responses, cerebral β-amyloidosis, and neuronal death. Conversely, treatment regimens associated with decreased peripheral levels of pro-inflammatory cytokines and sustained levels of the anti-inflammatory cytokine IL-10 promoted a microglia profile associated with dampened neuroinflammatory responses, increased amyloid-beta (Aβ) uptake and improved neuronal survival [34, 35]. Hence, microglia retain a long-lasting imprinting of peripheral inflammation.

A putative role of gut microbiota in the cross-talk between peripheral and central inflammation via microglia remains so far poorly assessed in physiological conditions. However, it has been reported that germ-free (GF) mice displayed global defects in microglia with altered cell proportions and an immature phenotype, leading to impaired innate immune responses [36]. Recolonization with a complex microbiota partially restored physiological features of microglia [36]. Remarkably, supplementation with SCFA mimics the effects of microbiota recolonization and is sufficient to reverse the alterations in microglial phenotype observed in germ-free mice [36]. SCFA, such as butyrate, induces functional changes in the microglia towards the expression of homeostatic (M0) phenotype and inhibits the production of pro-inflammatory cytokines following exposure to LPS both in vitro and in vivo [37]. Therefore, microbiota-generated metabolites can amplify beneficial feed-forward regulatory loops through their impact on peripheral-central immune cross-talk [37]. The underlying mechanisms likely may also include the capacity of microbiota metabolites, such as SCFA, to be transported across the BBB via monocarboxylate (MCT) and sodium-coupled monocarboxylate (SCMT) transporters [38] and thus subsequently influence the inflammatory response of microglia. In agreement, a recent study using differentiated HL-60 myelomonocytic cells to mimic immune functions of human microglia reported an altered production of pro-inflammatory cytokines/chemokines (IL-1β, TNFα and monocyte chemoattractant protein-1 [MCP-1]) in response to SCFAs [39]. If confirmed in vivo, these studies could provide a mechanism by which microbiota and derived metabolites may indirectly modulate neuroinflammation, including via amplifying deleterious feed-forward loops and modulating the peripheral-central immune cross-talk.

Of note, although this review focused on microglia as key innate immune cells of the brain and central player in neuroinflammation, increasing evidence support the instrumental contribution of astrocytes to neuroinflammatory responses, even if the available literature regarding the impact of microbiota on astrocytes is much less abundant than for microglia. It has been reported that LPS triggers astrocyte activation [40] whereas SCFAs inhibit LPS-induced astrocyte activation in vitro via NF-kB inhibition [41]. Remarkably, increased abundance of *Lachnospiraceae*, *Ruminococcaceae* and *Prevotellaceae* in the gut of NLRP3-deficient mice alleviated astrocyte dysfunction and depressive-like behaviors [42]. In addition, higher abundance of *Nitriliruptor*, *Youngiibacter*, *Burkholderia* and *Desulfovibrio* was found to correlate with astrocyte activation in some neurological disorders (e.g. in autism spectrum disorder) [43]. Besides, microbial metabolites of tryptophan have been shown to act in concert with endogenous type-I interferons in the CNS to modulate astrocyte activity and increase neuroinflammation [44]. Altogether, these data indicate that microbiota-mediated modulation of astrocyte reactivity may likely play a role in various neuroinflammation-associated neuropsychiatric and neurological conditions. Obviously, microbiota could also impact astrocytes indirectly, via microglia-astrocyte communication [45].

Overall, although the cross-talk between peripheral and central inflammatory responses is increasingly documented, the mechanisms underlying the modulation of microglia activation by microbiota are not completely elucidated. According to the current view, they may include indirect mechanisms such as passive transport of circulating lipophilic microbiota-derived metabolites across the BBB. Hydrophilic microbiota metabolites may also enter the brain from circulation at the specific anatomic locations where BBB is absent, such as for instance the circumventricular organs and choroid plexus. In addition, microbiota-generated metabolites and constituents can trigger pro-inflammatory mediator (e.g. cytokines) production by immune cells present in the gut sub-mucosal compartment of the lamina propria. These pro-inflammatory cytokines diffuse from the gut submucosal interstitial space via capillaries to reach the peripheral circulation and may in turn trigger circulating lymphoid and myeloid immune cells to produce additional cytokines. Circulation-born cytokines can reach the brain via cytokine transporters expressed by endothelial cells of the BBB and subsequently modulate cytokine production locally, notably by microglia and astrocytes. In the particular case of meningeal immunity, circulating cytokines can activate lymphoid and myeloid cells present in the meninges to secrete cytokines, which are transported into the brain parenchyma via the glymphatic system and yield brain-resident glia activation and subsequent brain-born cytokine production.

#### Direct actions via vagus nerve

In addition to the indirect mechanisms discussed in the previous section, direct pathophysiological mechanisms via primary autonomic afferents of the vagus nerve have been involved in the cross-talk between peripheral and central inflammation [46], though they remain much less studied.

A direct neural connection through the vagus nerve allows bidirectional brain-gut communication [4, 7]. Among the underlying mechanisms, the neuroactive microbiota-derived molecules can directly modulate vagus nerve output. The relevant microbiota products comprise numerous neurotransmitters, including dopamine, serotonin, norepinephrine and gamma-aminobutyric acid (GABA) [47]. In addition, microbiota-induced cytokine production via infiltrated or resident immune cells in the vicinity of gut vagus nerve terminals can also play a role in regulating vagus nerve output. Indeed, a study has directly demonstrated the capacity of TNFα and IL-1β to trigger neuronal activity in a cytokine-specific and dose-dependent manner along the vagus nerve [48]. The microbiota impact on vagus nerve activity is crucial with respect to the recently discovered neural control of the immune response in a reflex-like manner. The relevant “vagal immune reflex”, though beyond the scope of this review, involves the release of the acetylcholine neurotransmitter in response to vagus nerve stimulation, which by controlling the activity of immune cells dampens the inflammatory reaction [49].

However, immunomodulatory and neuromodulatory actions of microbiota are overlapping. Consistently, a very recent study reported that manipulating microbiota, by using antibiotics or GF adult mice, induced profound alterations in gene expression, not only in microglia but also in excitatory neurons of the medial prefrontal cortex, which was associated with defective neuronal encoding activity and learning-related post-synaptic remodeling of dendritic spines. Surprisingly, all these alterations were not correlated with neuroinflammatory changes and persisted even after the vagotomy, thus suggesting that microbiota metabolites may affect neuronal activity directly [50]. These discoveries open an exciting field of research focused on deciphering the causal relationship between microbiota metabolite-related neuronal and microglia alterations, which may help answering if microbiota metabolites could also impact microglia and neurons independently, either sequentially or concomitantly.

Thus, the central role of the gut-brain axis in (neuro)inflammation and neurodegenerative diseases is now increasingly recognized, with compelling evidence pointing to the involvement of microbiota in some of these disorders. Notably, in addition to AD, dysbiosis has also been associated with PD, MS and ALS (Background). The studies on putative involvement of microbiota in the pathogenesis of MS (513 hits in PubMed for “microbiota- Multiple Sclerosis” since 2010, as per December 2021), PD and AD (622 and 709 hits in PubMed since 2012 and 2013 for “microbiota-Parkinson” and “microbiota- Alzheimer”, respectively) are more numerous and slightly early than ALS studies (89 hits for “microbiota-Amyloid Lateral Sclerosis since 2014). Although there is currently no unifying concept on the involvement of microbiota in neurodegenerative diseases, the available evidence appears more consistant for PD (for recent review, see [51]) than for AD. However, with more than 200 publications per year since 2020, the impact of microbiota on AD is the one of the most dynamic fields of the research in the domain and the general hope is that this emerging data will help formulate a consensus. In the following sections we will specifically provide comprehensive and timely analysis of microbiota within the AD literature to illustrate recent advances pointing to the impact of microbiota on neuronal activity via peripheral modulation of inflammatory tone.

## Cognitive dysfunction and microbiota in Alzheimer’s disease

### Alzheimer’s disease: role of neuroinflammation

AD is an age-related and yet incurable neurodegenerative disease with still poorly understood etiology. Clinically, AD is currently diagnosed late in the course of the disease, decades after the pathology has begun. It is the most common cause of dementia and the number of affected people is rapidly increasing, making it a major public health concern. The number of patients with dementia worldwide was estimated at 50 million in 2020, and this prevalence is predicted to double every 20 years, reaching 82 million in 2030 and 152 million in 2050 [52].

Patients display progressive memory impairments and cognitive decline that correlate with synaptic dysfunction and neuronal loss. These alterations manifest in two early stages: Subjective Cognitive Decline (SCD) and Mild Cognitive Impairment (MCI). The first one is clinically undetectable, manifests as minor distractions and occurs during the preclinical stage. SCD can progress to the prodromal stage of AD (MCI due to AD) that is clinically quantifiable and currently the earliest stage at which AD is diagnosed [53]. AD is characterized by several biological hallmarks including the accumulation of two pathological protein species: Aβ peptide and hyperphosphorylated tau, which aggregate into extracellular amyloid plaques and intracellular neurofibrillary tangles (NFT), respectively [54]. The accumulation of these neurotoxic species starts several years and even decades before the onset of clinical symptoms, when the diagnosis cannot be established yet. Notably, Aβ reduction in the CSF (due to the deposition of plaques in the brain parenchyma) precedes tau accumulation and is detected up to 15 years or more before clinical diagnosis [55].

Using animal models, it has been shown that accumulation of Aβ in the brain parenchyma, known as amyloidosis, can disrupt neuronal signaling, drive synaptic and neuronal loss, and progressively impair cognitive function [3, 56]. Amyloid deposits and NFT also trigger a chronic innate neuroinflammatory response as reflected by activation of surrounding microglia and astrocytes [2, 57]. Microglial cells are activated by Aβ peptides and pathological Tau species that bind to several PPRs such as TLRs and scavenger receptors (SRs) [58]. This translates into microglia activation [35, 58], which is in turn associated with altered expression of phagocytosis-related genes, including CD33, CR1 and Abca7 related to cerebral Aβ-clearance in the context of AD pathology [58]. Of note, genome-wide association studies (GWAS) have identified single-nucleotide polymorphisms in such genes, which are associated with a higher risk to develop AD [59]. Interestingly, apolipoprotein E (ApoE), which is the strongest genetic risk factor for AD, plays a role in the shift from the homeostatic M0 to DAM/MGnD phenotype, by activating the triggering receptor expressed on myeloid cells-2 (TREM2). In the brain, TREM2 is expressed specifically by microglia and is tightly associated with neuroinflammation [29, 30].

AD-related chronic neuroinflammation at the advanced stages of pathology manifests by microglial production of pro-inflammatory cytokines/chemokines, including IL-1β, IL-6, TNFα, CC-motif chemokine Ligand-5 (CCL5), Macrophage Inflammatory Protein-1α (MIP-1α) and MCP-1 via TLR4, TLR2 and inflammasome NALP3 signaling [31, 33, 58]. However, enhanced levels of cytokines such as TNFα are already detectable at early stages of AD in both humans [60] and animal models of AD-like pathology [61]. The complement system is also involved in the clearance of Aβ plaques in the early stages of the disease and plays a versatile role in AD according to disease stages [62, 63]. The complement component 1q (C1q), for instance, has a neuroprotective role in the early stages of AD but acquires a neurotoxic role as the disease progresses, by contributing especially to the activation of microglia and its shift towards pro-inflammatory phenotype [63]. Interestingly, studies have shown that the complement system promotes neurogenesis in the dentate gyrus, a brain area that has a key role in hippocampal memory formation, which is compromised in AD [62]. Conversely, when acting in combination with interleukin-1 alpha (IL-1α and TNFα, C1q promotes the differentiation of reactive astrocytes towards an A1-like neurotoxic functional profile [64]. Furthermore, C1q triggers early synaptic loss in AD-like mouse model of amyloid pathology, via C3- and CR3-mediated synaptic pruning involving microglia [65]. Besides, C3 mediates (at least in part) hippocampal synapse loss due to aging and amyloid deposition in wildtype (WT) and APP/PS1 mice, respectively. Genetic deletion of C3 protected hippocampal synapses and cognition in both WT and transgenic mice despite increasing plaque deposition in the APP/PS1 mouse model [66, 67].

As disease progresses, excessive and chronic neuroinflammatory signaling leads to neural and glial cell death [29, 65]. Overall clearance of Aβ becomes compromised while microglial production of pro-inflammatory mediators remains elevated. This yields neurodegeneration and further feeds neuroinflammation along a vicious cycle [68]. Neuronal loss is also accelerated by the release of ROS and NOS by microglial cells [33].

At advanced stages of AD, peripheral macrophages may also be recruited to the brain in an effort to clear the plaques as microglia clearance capacity decreases, although this point is still debated [69–71]. At these advanced stages, infiltration of peripheral immune cells is likely facilitated by the altered BBB integrity and permeability, further exacerbating inflammatory changes in the CNS during AD [69]. In addition, peripheral inflammation contributes to AD progression and is correlated to cognitive deficits, specifically at MCI stage, and increased peripheral production of IL-1β and TNFα is associated with a higher risk of AD [69, 72]. Interestingly, previous studies suggested that non-steroidal anti-inflammatory drugs may reduce the incidence of AD development in long-term users, particularly when the treatment is applied early, e.g. in pre-symptomatic patients, but no beneficial effect could be evidenced when administered later, i.e. in symptomatic patients [71]. This suggests that different immune processes occur during each stage of the pathology [10, 71] and that early stage AD-associated neuroinflammation may even be beneficial [73].

Altogether, recent experimental evidence points to an intricate interplay between central neuroinflammation, peripheral inflammation and AD-related cognitive dysfunctions.

### AD-related impairment of synaptic excitability and plasticity: focus on LTP and link with neuroinflammation

**Fig. 1:**
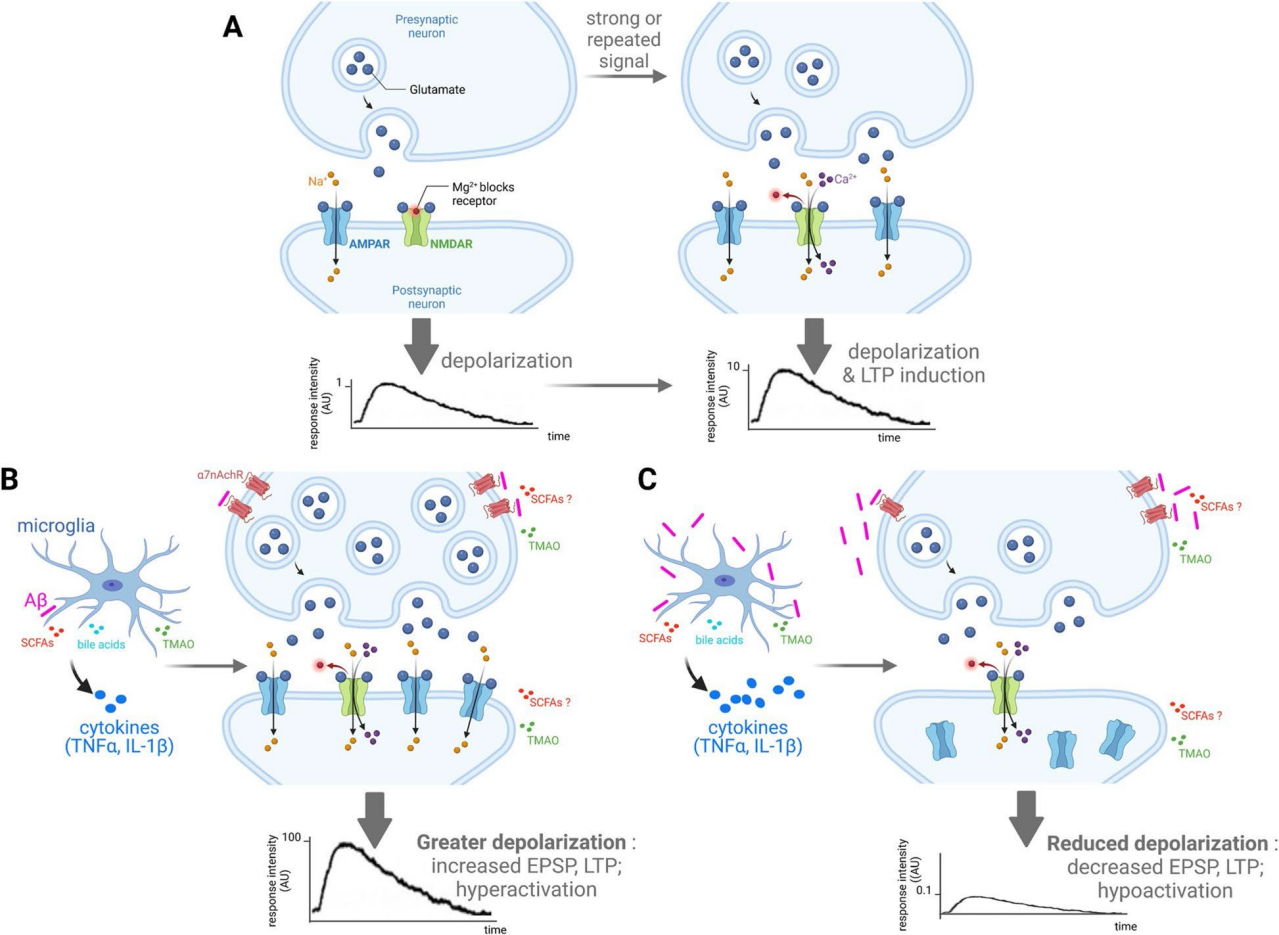
Induction and expression of LTP in physiological and AD-related pathological conditions in animal models of AD-like pathology and its modulation by microbiota products. (**A**) In physiological conditions, optimal input to pre-synaptic hippocampal neurons triggers the release of glutamate into the synaptic cleft. This activates membrane AMPARs on the post-synaptic neurons and allows the entry of Na^+^ into the cell, yielding a excitatory postsynaptic potential (EPSP). When tetanic stimulation is given, a large amount of glutamate is released from presynaptic terminals, which increases the EPSP produced by postsynaptic membrane, resulting in the removal of Mg^2+^ blocked in NMDAR and its subsequent activation. NMDAR activation allows the influx of Ca^2+^ as well as Na^+^ ions into the cell, leading to the activation of Ca^2+^-calmodulin-dependent protein kinase-II, which phosphorylates AMPAR, increases its conductivity, promotes the transfer of AMPAR from the cytoplasm to the postsynaptic membrane and increases its density, resulting in LTP. (**B**) Early stages of AD-like pathology are likely associated with elevated glutamate release by pre-synaptic neurons and increased glutamate concentration in and around the synaptic cleft. Indeed, Aβ can stimulate glutamate release through α7nAchR activation on the pre-synaptic neuron and contribute to pre-synaptic facilitation. Pro-inflammatory cytokines, such as TNFα and IL-1β, produced by microglia in the presence of accumulating Aβ, can upregulate AMPAR expression on postsynaptic membrane, which allows higher ion influx and a greater depolarization. All of these changes at the synaptic level lead to a neuronal hyperactivation in the hippocampus and increased LTP in the early stages of AD. Microbiota metabolites such as SCFA, BA and TMAO have been identified in the brain (for details, see Impact of microbiota on neuroinflammation and AD and gut dysbiosis: link with neuroinflammation and involvement of microglia). The negative impact of TMAO on synaptic plasticity (i.e. LTP impairment) has been reported [76] but as the relevant experiments were carried on hippocampal slices *ex-vivo* which were incubated in the presence of TMAO, it is not clear presently whether TMAO exerts its effect directly on neurons, or indirectly by acting on microglia and subsequently affecting neuronal function via microglia-neuron cross-talk. In contrast, in the 5XFAD mouse model, sodium butyrate (one of the SCFAs) promoted synaptic plasticity as assessed by LTP electrophysiology recording in vivo [77]. The experimental set-up used in this study was unable to determine whether the observed impact on neuronal activity is direct or rather indirect (via microglia). There is currently no data on the putative impact of BA on neuronal activity. (**C**) Later stages of AD-like pathology are characterized by hypoactivity of the glutamatergic neurotransmission as a consequence of the paradox hyperactivity in earlier stages. Elevated Aβ levels yields reduced glutamate release by pre-synaptic neurons through inhibition of α7nAchRs, which reduces post-synaptic activation of NMDARs. Moreover, chronic stimulation of AMPARs during early stages of AD pathology leads to desensitization and internalization of these receptors. In addition, excessive production of pro-inflammatory cytokines (e. g. TNFα, IL-1β…) by microglia due to toxic conditions created by continued Aβ accumulation, can trigger neuronal death and release of glutamate from dying neurons, thus escalating neurotoxicity. Altogether, this results in reduced LTP and EPSP (and a mirror increase in LTD, not depicted in the figure for the sake of clarity) that are associated with cognitive deficits. Created with Biorender.com

According to the current hypothesis of AD pathogenesis, synaptic dysfunctions precede the onset of cognitive impairment [74]. Alteration of synaptic activity occurs already during the pre-symptomatic stage of AD and has been associated with an increased glutamatergic tone and excitability of pyramidal neurons in vulnerable brain regions, notably in the hippocampus. An increase in glutamatergic transmission yields hyperexcitability and could result either from a decrease in inhibitory GABAergic neurotransmission, or directly from the increased efficiency of glutamate. The mechanisms of hyperexcitability based on the increased glutamatergic transmission involve α-Amino-3-hydroxy-5-methyl-4-isoxazolepropionic acid (AMPA) and N-methyl-D-aspartate (NMDA) types of ionotropic glutamate receptors: AMPA receptors (AMPARs) and NMDA receptors (NMDARs), respectively. Hyperexcitability translates into increased AMPAR-mediated response during basal synaptic transmission as reflected by increased excitatory post-synaptic potential (EPSP), at least in the hippocampus [75] (Fig. 1).

Impaired hippocampal GABA receptors (GABARs) transmission also contributes to the hyperexcitability characteristic of early AD pathology, at least in animal models. In line, loss of GABAergic neurons is observed in all hippocampal regions except the subiculum in a murine AD-like model [78], and is detectable from the early stages of the disease [79]. Interestingly, in this AD-like mouse model (TgCRND8), GABAergic neurons appear to be affected before glutamatergic or cholinergic neurons [78, 79].

The imbalance between neuronal network inhibition and excitation yields aberrant excitatory activity and synchronization, and increases susceptibility to seizures [78, 79]. These synaptic dysfunctions result in impairments in learning and memory circuits, which manifest as an attenuated long-term potentiation (LTP) and an increased long-term depression (LTD), both tightly related to synaptic plasticity. LTP is experimentally induced by repeated high-frequency stimulation yielding an enhancement of the transmission efficiency between synapses. It is now well recognized that in the presence of Aβ, there is a failure or aberrant LTP induction resulting in decreased synaptic plasticity via glutamate excitotoxicity [80]. On the other hand, the transmission efficiency between synapses decreases in low-frequency stimulation protocols aimed at LTD induction, the latter being also impaired in the presence of Aβ. Abnormal LTP/LTD are likely related to Aβ-mediated enhancement of NMDARs signaling, reduced expression of AMPARs on the post-synaptic membrane, attenuated GABAergic inhibition and/or impairment of presynaptic and postsynaptic calcium channels [81]. Resulting deficits in hippocampal LTP in both murine AD-like models [82] and in human-derived synaptosomes [83] together with impaired LTP in animal models of aging [82, 84] are in line with the progressive feature of cognitive dysfunctions in AD. Age-related deficit in LTP is also correlated with the accumulation of ROS and neuroinflammation, particularly the increase of IL-1β [74, 82, 85, 86] and TNFα [61, 72, 86]. Consistently, it was shown that IL-1β and TNFα impair LTP induction in both CA1 and dentate gyrus areas of the hippocampus [87]. Moreover, the orchestrated regulation of glutamate and GABA receptors, which are respectively increased and decreased by cytokines TNFα and IL-1β [72], can further enhance LTP/LTD impairments.

### AD and gut dysbiosis: link with neuroinflammation and involvement of microglia

At advanced stages of the disease, AD-like murine models and patients with AD display a gut microbiota dysbiosis characterized by changes in microbial diversity and composition, with increased abundance of pro-inflammatory taxa and decreased abundance of anti-inflammatory taxa.

#### Comparison of AD- and age-related dysbiosis in human and rodent models

In patients with clinical symptoms of AD, a previous report revealed a decrease in *Firmicutes* phylum [88], similar to the alterations seen in inflammatory conditions like inflammatory bowl diseases [89]. A recent study reported a reduction of *Faecalibacterium prausnitzii*, an anti-inflammatory bacterium (belonging to the *Firmicutes* phylum), in patients with MCI compared with healthy subject [90]. Microbiota changes also consists of an increase in *Bacteroidetes* phylum, which correlates with an increase in CSF levels of chitinase-3-like protein 1 (YKL-40), a marker of microglial activation [88]. Increased abundance of Gram-negative intestinal bacteria such as *Bacteroides* in AD patients may result in increased LPS translocation from the gut to the systemic circulation, which may contribute to AD pathology through the stimulation of systemic inflammation. Additional studies have confirmed AD-related decreases in microbiota richness and diversity and clearly evidence of different compositions in patients when compared to cognitively normal controls or MCI (reviewed in [91]). Yet, since the two pioneering clinical studies of microbiota in AD, performed in USA [88] and Italy [92] in 2017, an additional study from USA [93] and six studies from China [94–99] have been published. Remarkably, but not very surprisingly, these recent studies pointed to striking differences in terms of AD-associated microbiota composition depending on geographical and ethnical factors. For instance, the second study from USA [93] did confirm the increase in *Bacteroides* in AD patients, as reported in the pioneer study from the same country [88], while the opposite was found in a Chinese study [95]. Similarly, *Actinobacteria* was found increased [88] and decreased [96] in studies from USA and China, respectively whereas *Bifidobacterium* was in contrast decreased in an American study [88] and increased in Chinese cohort [97]. These differences stress the importance of considering environmental factors such as geographical and ethnic origin (and hence related differences in diet) while studying the alterations of microbiota in AD patients, and more generally in global populations. The observed differences should also foster additional studies of AD-related dysbiosis at the regional and country level, before any generalization at the international level can be drawn. This is of utmost importance in the perspective of targeting microbiota as a putative future therapeutic approach (see Restoring AD-associated neuronal function by targeting microbiota?).

Despite the above discussed geographical differences, dysbiosis in AD patients has been convincingly correlated with pathological outcomes. For example, increase in pro-inflammatory *Escherichia / Shigella* genera and decrease in anti-inflammatory *Eubacterium rectale* have been correlated with reduced CSF Aβ_42_/Aβ_40_ and increased phospho-tau and phospho-tau/Aβ_42_ levels [92]. Furthermore, an increase in circulatory biomarkers of inflammation such as IL-1β, NLRP3 and CXCL2 was positively- and negatively-correlated with increased *Escherichia / Shigella* and decreased *E. rectale* respectively, pointing to the capacity of microbiota to drive peripheral inflammation [92].

Because the major risk factor for AD is age, harnessing the age-related alterations of microbiota may turn out to be informative for deciphering the mechanisms linking gut dysbiosis and AD pathogenesis. A seminal study of an Italian cohort comparing microbiota between young adults, older adults, and centenarians has revealed that the oldest-old exhibited a much more pro-inflammatory microbiota than the younger people [100]. Consistently, another study reported that the gut microbiota of the elderly in an Irish cohort is substantially different from the younger adults, with a loss of diversity and a shift towards a pro-inflammatory phenotype [101]. In line, the proportion of *Firmicutes* (butyrate-producing bacteria) is significantly lower in older individuals in comparison to younger adults where *Firmicutes* outnumber *Bacteroidetes* [102]. Nevertheless, some inconsistencies still remain regarding the evolution of the number of *Bacteroidetes* in the course of aging [103]. Moreover, the levels of microbial SCFAs decrease in the course of aging [104] whereas SCFAs remain abundant in centenarians [104] suggesting that SCFAs may be protective against aging.

Considering transgenic AD-like mouse models, gut dysbiosis is detected at advanced stages of the pathology. Hence, 8 months-old APPPS1 mice display a significant reduction in *Firmicutes, Verrucomicrobia, Proteobacteria* and *Actinobacteria* phyla, and a significant increase in *Bacteroidetes* and *Tenericutes* phyla as compared to age-matched wild-type controls [105].

Regarding alterations of microbiota composition along the progression of AD-like pathology, a few studies reported that some microbial strains evolve between the pre-symptomatic and symptomatic stages of the pathogenesis, while other strains are continuously present from the pre-symptomatic stage and persist all along the pathogenesis. For instance, a significant increase in *Lactobacillus* is observed in Tg2576 mice during the progression towards the symptomatic stage, while a significant reduction in *Ruminiclostridium* found in pre-symptomatic Tg2576 mice persists later in the symptomatic stage [106]. Two other studies confirmed some of the aforementioned pre-symptomatic stage findings in additional murine models, notably the increase in *Lactobacillus* in 3xTg-AD [107] and decrease in *Ruminiclostridium* in APP/PS1 [108]. A more recent study reported that the decrease in the relative abundance of *Firmicutes* and *Bifidobacteria* and the increase in the relative abundance of *Bacteroidetes*, already present at 5 months of age in 5xFAD mice (i.e. during the pre-symptomatic stage in this model) in comparison to non-transgenic controls, persist throughout the course of aging. Indeed, analogous alterations are still evident at the overt stage of pathology (15 months of age). Consistently, this study found reduced *Firmicutes* / *Bacteroidetes* ratio in the gut of 5xFAD mice at both ages (5 and 15 months) concurrently with increased NLRP3 inflammasome and IL-1β production, which were positively correlated with astrogliosis and microgliosis along with increased cerebral NLRP3 inflammasome and IL-1β [109].

In addition, a longitudinal approach was also used to assess how aging affects the establishment of dysbiosis in two different AD-like mouse models. In the APP/PS1 model of amyloidosis, studies at 3-, 6- and 24-months of age (corresponding in this model to pre-symptomatic stage, onset and overt stages of pathology, respectively) pointed to the absence of dysbiosis at 3 months whereas dysbiosis was manifest in 6-months old APP/PS1 mice. Accordingly, by 6 months of age, pro-inflammatory phyla *Proteobacteria* and *Erysipelotrichaceae* increased in APP/PS1 mice compared to controls [110]. Importantly, this work reported that in addition to the genotype effect, an aging effect was also prominent. Along the aging process, *Turicibacteriaceae* and *Rikenellaceae* increased in both APP/PS1 and control mice, although *Bacteroidetes* remained stable [110]. In a similar study using P301L tau transgenic mice to model the tau pathology, a significant change in both diversity and composition of microbiota was observed. *Firmicutes* and *Actinobacteria* were decreased whilst *Bacteroidetes* was increased in P301L mice, starting at pre-symptomatic stage (3 months) in comparison to control mice. By contrast, *Tenericutes* was decreased only in P301L mice at the overt stage of pathology (i.e. 10 months in this model) [111].

However, in rodent studies of aging-related microbiota alterations, the pattern of change was inverted in comparison to humans, thereby suggesting that it may be species-specific. Abundance of *Firmicutes* was higher whilst the abundance of *Bacteroidetes* was lower in old (15 months) *versus* young (2 months) old C57Bl6 mice [112]. These microbiota alterations in old mice were concomitant with increased pro-inflammatory (TNFα, IL-1β and IL-6) cytokines expression in the plasma, gut and brain, concurrently with an increased level of LPS both in the plasma and brain, increased cerebral expression of Iba-1, TLR4 and nuclear translocation of NF-κB pointing to microglial activation [112]. An analogous study in old (20–24 months) *versus* young (3 month-old) Sprague–Dawley rats reported a similar change in microbiota, with lower relative abundance of *Bacteroidetes* in aged compared to young rats, and conversely higher relative abundance of *Firmicutes*. The ratio of *Firmicutes* / *Bacteroidetes* increased several fold with aging. As in mouse studies, this age-related microbiota alteration was correlated with increased serum and hippocampal levels of pro-inflammatory (TNFα, IL-1β and IL-6) cytokines in old rats [113]. The observed changes in microbiota was further correlated with cognitive impairment and decreased neuronal activites in resting state-fMRI [113]. Fecal microbiota transplantation (FMT) approach (see discussion on FMT below) using young rats as recipient and old rats as microbiota donor indicated that FMT reshaped gut microbiota of young rats towards that of old rats, leading to impaired cognitive behavior in young recipient rats, as well as decreased neuronal activity in resting state-fMRI, deteriorated structural and morphological synaptic characteristics and increased levels of pro-inflammatory cytokines in serum and hippocampus [113]. This study thus demonstrated a causal relationship between an age-related shift in gut microbiota and neuroinflammation, neuronal dysfunction and cognitive impairment. Accordingly, when the opposite approach was used in mice, i.e. FMT of young mice microbiota to old recipient, it counteracted age-related hippocampal neuroinflammation, metabolome and transcriptome alterations, and rescued selective cognitive deficits [114].

It has to be stressed that comparison between the reported phyla alterations in rodent models remains difficult since the correspondence between staging of AD pathogenesis among different models is not standardized yet [106–108]. Moreover, how these age- and AD-related microbiota alterations interact mutually remains to be further explored. Besides, although dysbiosis appears as a common trait of AD pathogenesis in both human and rodents, different specific phyla might likely be affected in a species-specific manner. Multi-national and longitudinal studies in human, even if difficult to set up, will be required to expand our knowledge on AD- and age-related dysbiosis.

#### Role of microbiota-related permeabilization of gut and brain barriers

The alterations in the gut microbiota composition can lead to increased permeability of both the intestinal-blood barrier (“leaky gut”) and the BBB (“leaky brain”) [6, 115]. Indeed, the gut mucosa layer protects the host from pathogen infiltrations and the composition of the gut bacteria determines its properties. Whereas some bacterial strains that contribute to the preservation of the intestinal barrier integrity are reduced in AD patients (*Akkermansia muciniphila*, *Bifidobacterium infantis*), other strains that disrupt the epithelial cells integrity are increased (*Helicobacter pylori*, *Shigella*, *Escherichia coli*) [115, 116]. Moreover, it has been reported that epsilon-4 allele Apolipoprotein E (ApoE4) carriers, which are at much higher risk to develop AD [3, 29, 68], display a lower abundance of protective SCFA-producing bacteria such as *Ruminococcaceae*, making them more vulnerable to loss of intestinal integrity and increased permeability [116]. In addition, due to permeabilization of the gut barrier, AD dysbiosis-related gut microbiota products, such as β-N-methylamino-L-alanine (BMAA), LPS and microbial amyloid proteins, may reach the circulation and enter the CNS, especially if BBB is permeabilized (see below) to promote neurodegeneration, cognitive impairments, astrogliosis, accumulation of NFT and cerebral amyloidosis [6]. In particular, microbial amyloid proteins produced in the gut, such as curli, may cross-seed cerebral Aβ-aggregation in a prion-like manner and subsequently prime inflammatory response both in the brain and in the periphery [117].

Gut barrier dysfunction can be instrumental to “leaky brain”, as it yields translocation of pro-inflammatory bacteria-derived endotoxins and metabolites into the bloodstream, which could contribute to altering the permeability of the BBB. The related decreased BBB integrity may then contribute to promote innate neuroinflammation and neurodegeneration [115, 116]. Certain gut-produced metabolites that could influence neurodegeneration and AD pathology are capable of infiltrating the brain, like the microbial-derived metabolite trimethylamine N-oxide (TMAO) and bile acids [116] (Fig. 1). Indeed, metabolomics analysis comparing bile acids measured in post-mortem brain samples from AD patients and cognitively normal individuals showed an association between cognitive decline and increased amounts of secondary bile acids (such as deoxycholic acid and lithocholic acid), which are produced by the gut microbiota from primary bile acids [118]. In addition, brain amyloid deposition is positively associated with circulating LPS but also acetate, valerate and propionate, and negatively associated with circulating butyrate [119].

#### Interventional approaches and interplay of gut dysbiosis / neuroinflammation / AD

To understand the mechanisms linking gut dysbiosis to AD pathogenesis, another useful approach consists in assessing the consequences of altering the gut microbiota in early life, which is a crucial period for the effects of gut microbiota on host functions. Broad-spectrum antibiotics cocktails applied in two mouse models of AD-like amyloidosis (APP/PS1 [120] and APPPS1 [121]) yielded a profound alteration of the microbiota composition, notably of *Lachnospiraceae* and *S24-7* genera [121], and a significant decrease in cerebral amyloidosis. Antibiotic-treated mice displayed elevated levels of Tregs both in the blood and brain, while no difference was observed in Th1, Th2 and Th17 CD4 + T-cell subsets. Immune activation was also evidenced by increased blood levels of IL-1β, interleukin-2 (IL-2), interleukin-3 (IL-3), CC-motif chemokine Ligand-11 (CCL11) and Stem Cell Factor (SCF), as well as decreased IL-6 levels [121]. Immune profiling of CSF, which reflects the immune milieu of the brain, indicated a decrease in IL-2, IL-3 and SCF, without alteration in IL-1β, CCL11 and IL-6 expression [121]. Of note, the effect of antibiotics was likely impacted by hormones as the phenotype was different in males and females. A decrease in circulating pro-inflammatory (IL-1β, IL-2, IL-3, IL-6, CCL11, CCL5, CXC-motif chemokine Ligand-5 (CXCL5) and SCF) and an increase in anti-inflammatory (e.g. IL-10) cytokines/chemokines were reported in male APPPS1 mice, while the opposite was observed in females [122]. In addition, Aβ-related microglial reactivity was significantly reduced in antibiotic-treated mice, as documented by a decreased accumulation of microglia around Aβ plaques and their morphological alterations, i.e. thinning of the cell bodies and processes. This was particularly observed in male mice, where the antibiotic treatment prevented the transition from homeostatic M0 towards DAM/MGnD transcriptomic profile, concomitantly to alterations in specific microbial genera, such as *Allobaculum* and *Akkermansia* [122].

As already discussed, microglia play a central role in translating microbiota actions from peripheral to central inflammatory changes, which under some circumstances may be beneficial in AD. Indeed, the absence of microbiota in GF (axenic) APPPS1 mice was demonstrated to reduce both microgliosis and cortical production of pro-inflammatory cytokines, and to delay disease progression [105]. The absence of microbiota leads to defects in microglia numbers and maturation phenotype in both germ-free wild type [36] and APPPS1 mice [105]. Remarkably, Aβ pathology worsens when GF APPPS1 mice are colonized with microbiota from either APPPS1 or conventional mice, and microglial phenotype is altered accordingly [105].

Although beyond the scope of this review, the impact of microbiota on other neurodegenerative disorders shares both similarities and differences with the alterations seen in AD. Most studies reported that PD dysbiosis involves increased abundance of *Enterobacteriaceae* and decreased abundance of *Bacteroidetes, Lachnospiraceae* and *Prevotellaceae* [123, 124]. As discussed in the beginning of AD and gut dysbiosis: link with neuroinflammation and involvement of microglia, this is in contrast with decrease in *Firmicutes* and increase of *Bacteroidetes* phylum, reported in AD [88]. However, by analogy to Aβ in AD, gut microbiota is required for α-synuclein aggregation, microglia activation and induction of TNFα and IL-6 in vulnerable brain areas [125]. Dysbiosis in PD patients is specifically associated with reduced cellulose-degrading bacteria *Blautia*, *Faecalibacterium* and *Ruminococcus* [126]. Animal studies in which GF mice over-expressing human α-synuclein were supplemented by a mixture of SCFA elegantly confirmed the crucial role of SCFA in triggering neuroinflammation in PD pathogenesis [125], which contrast with anti-inflammatory actions SCAFs likely exerts in the cours of aging [104] (AD and gut dysbiosis: link with neuroinflammation and involvement of microglia). Regarding MS, increases in *Akkermansia muciniphila* and *Methanobrevibacter smithii,* and a decrease in *Butyricimonas* have been reported in patients in remission compared to the healthy controls [127]. Similar to alterations in AD and PD, MS patient-derived *A. muciniphila* triggers a pro-inflammatory response, both in vitro (in human peripheral blood polymorphonuclear cells) and in vivo (after colonization in GF mice). Conversely, *Parabacteroides distasonis,* which is less abundant in MS patients than in healthy subjects, triggers anti-inflammatory IL-10 expressing-Tregs [128].

**Fig. 2:**
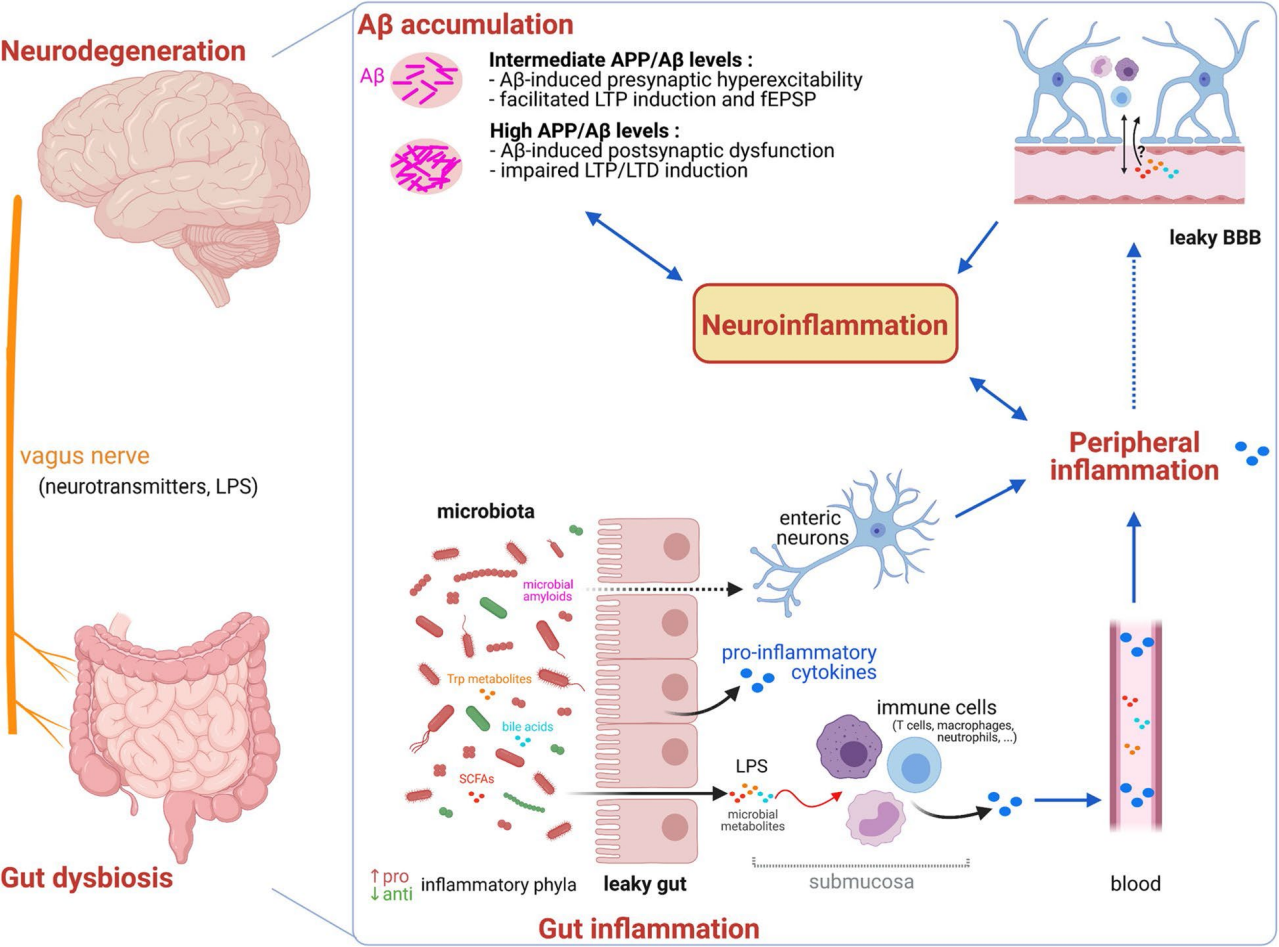
Hypothetical link between gut dysbiosis and mechanisms leading to the pathogenesis of AD. Alterations in the gut microbiota composition and function in AD patients increases permeability of the intestinal barrier and likely BBB, which creates a vicious cycle of enhancing inflammation at the gut and the CNS level. Early stages of AD (low concentrations of Aβ) are characterized by increased excitability of pyramidal neurons in the hippocampus subsequent to the increased glutamatergic neurotransmission, which in turn translates into presynaptic facilitation, enhanced fEPSP and LTP. Conversely, later stages of AD (high concentrations of Aβ) are associated with marked decrease in excitability and fEPSP, as well as reduced LTP and enhanced LTD, likely related to a decrease in the number of synaptic AMPA receptors and progressive memory loss. Created with Biorender.com

Altogether, concordant data highlight the influence of gut microbiota on the development of pathology in neurodegenerative diseases in general and of amyloid pathology in AD, in particular. The accumulated data point to the link between microbiota and neuroinflammation via microglia modulation [129] (Fig. 2), including during the pre-symptomatic latent period of the pathogenesis, at least in AD. Besides, according to a recently emerged new concept, it is important to keep in mind that AD should be considered not just as a neurological- but rather as a systemic disease [10, 130]. As already discussed (Alzheimer’s disease (AD): role of neuroinflammation), according to this new concept, inflammation in AD is not restricted to neuroinflammation but also encompasses the systemic inflammation with a sustained interaction between the peripheral, systemic and CNS inflammatory compartments [10], and AD pathology is often associated with a number of cardio-vascular, hepatic, renal, metabolic and other systemic dysfunctions [130]. Of utmost importance, these additional systemic abnormalities may possibly involve impairment of peripheral metabolism of Aβ, such as its peripheral clearance by, for instance, uptake, phagocytosis or endocytosis via monocytes, erythrocytes, lymphocytes, neutrophils and hepatocytes [130]. Although it is currently unknown how gut microbiota might affect such peripheral clearance of Aβ from the circulation, the impact of microbiota on neutrophils has been well documented [131]. Answering these questions is critical as considerable amount of Aβ is produced in the periphery [130] and such peripheral Aβ, including from gut [132], can reach the brain. Future studies will certainly help uncover whether Aβ-related alteration of gut microbiota is causal to inflammation / neuroinflammation, or if gut dysbiosis is a consequence of Aβ-induced inflammation / neuroinflammation. Of note, these two possibilities are not exclusive and might both be part of a vicious cycle that fosters AD pathogenesis.

## Putative interventions aimed at improving neuronal activity via microbiota-mediated immunomodulation

### Restoring AD-associated neuronal functions by peripheral immunomodulation

During the last decade, early peripheral immunomodulation in pre-symptomatic rodent models of AD-like pathology convincingly demonstrated significant impacts on various hallmarks of the disease, including LTP impairments. Inhibition of peripheral TNFα (via subcutaneous injection of antagonist) before the onset of cognitive symptoms restores neuronal excitability and plasticity in TgCRND8 mouse model, and these effects persist even during the symptomatic stage [61]. This treatment also rescues impaired LTP and decreases activated immune cells in the brain, including infiltrated CD4 + T cells [133]. Similarly, early inhibition of TNFα in 3xTgAD mice prevents cognitive impairment, concomitantly reduces CNS infiltration of peripheral blood leukocytes [134] and reduces Aβ-plaque formation [135, 136]. Such treatment also reduces other neuropathological hallmarks in 3xTgAD mice, including microglia activation, phosphorylated tau protein and APP accumulation, and preserves synaptic function [135]. Interestingly, deletion of TNFα type-1 death receptor (TNFR1) reduces cognitive deficits in APP23 mice by improving learning and memory [137].

Similar results were observed when IL-1β is inhibited in AD-like mouse models. Blocking IL-1 receptor in 3xTgAD mice rescues cognitive impairments and attenuates microglial activation and tau pathology [138, 139]. IL-1 receptor antagonist (IL-1Ra) can even reverse LTP impairment caused by IL-1β [140]. Conversely, overexpression of IL-1β exacerbates tau phosphorylation and NFT formation, resulting in impaired LTP and memory [138]. These observations may be related to the inhibitory effects of IL-1β on the amplitude of NMDAR-mediated EPSP, by analogy to the previously reported effect exerted by another pro-inflammatory cytokine (interleukin-18 [IL-18]) on EPSP [140]. In addition, IL-18 can impair the induction of LTP in the dentate gyrus of Wistar rats and antagonists of IL-18 attenuate LTP and EPSP depression caused by this cytokine [140]. Besides, early peripheral immune modulation by selective amplification of Treg cells via peripheral administration of low-dose IL-2 impacts microglial response and delays the onset of cognitive deficits, improving cognitive functions at advanced disease stages in APPPS1 mice [141].

### Restoring AD-associated neuronal dysfunctions by targeting microbiota?

Targeting the gut microbiota and restoring its balance could represent a promising therapeutic strategy in AD. Several approaches can be considered, such as prebiotics (food components, typically non-digestible fiber compounds, with the capacity of inducing growth or activity of beneficial microorganisms), probiotics (live microorganisms which confer a health benefit on the host when administered in adequate amounts), or FMT, which consists in transferring a solution of fecal matter from a healthy donor to restore the gut microbiota of a patient suffering from a microbiota-related diseases) [142, 143].

#### Pro- and prebiotics approach

Regarding animal models, in spite of a convincingly demonstrated cross-talk between i) gut microbiota perturbation and peripheral immunomodulation (Impact of microbiota on peripheral inflammation/ immunity and  Impact of microbiota on neuroinflammation), ii) peripheral inflammation and neuroinflammation [7, 26, 27, 34] and iii) neuroinflammation and neuronal activity [48], the impact on synaptic activity of modulating microbiota has not been explored so far, particularly when considering the pre-symptomatic changes in AD.

To the best of our knowledge, except for one report (see below), all published evidence in this context come from studies carried out at advanced pathological stages. For instance, probiotics mixture (VSL#3) supplementation was applied to modify microbiota in non-transgenic aged male Wistar rats, used to mimic the age-associated features of AD-related neuronal dysfunctions, such as deficits in LTP and neurogenesis. This study reported attenuated age-related deficits in LTP, reduced neuroinflammation and decreased expression of specific markers of microglial activity such as CD68 and CD11b, which correlated with changes in microbiota composition following probiotic treatment [144]. This probiotic treatment was also associated with a significant increase in brain expression of genes associated with neural plasticity, like synapsin and Brain-Derived Neurotrophic Factor (BDNF), the latter being positively correlated with LTP [84]. The unique study that assessed the microbiota impact on LTP/LTD during the pre-symptomatic stage, using the 5xFAD model, reported that treatment with SCFA (e.g. sodium-butyrate) could ameliorate synaptic impairments at 2 months of age (corresponding to the pre-symptomatic stage in 5xFAD mice) via inhibition of neuroinflammation [77] which is in line with anti-inflammatory potential of SCFAs in “successful” aging [104] but contrasts pro-inflammatory effects of SCFAs in PD [125]. It was recently shown that administration of *F. prausnitzii* strains (pasteurized or live) isolated from healthy subjects improves cognitive impairment in an i.c.v. Aβ-injected mouse model, suggesting that *F. prausnitzii* could be a promising candidate for prevention of MCI [90]. Further studies in additional AD-like mouse models are needed to confirm the putative beneficial impact of *F. prausnitzii* on AD pathogenesis and to decipher the mechanisms by which it may modulate cognitive functions.

The improvement of LTP observed following probiotic administration could be attributed to changes in AMPAR/NMDAR ratio that are involved in excitatory glutamate synaptic transmission in the hippocampus, although this mechanism has not been addressed yet directly in AD-like models. Treating cognitively non-impaired middle-aged Sprague–Dawley male rats with probiotic (*Enterococcus faecium*) and prebiotic (inulin) supplementation for 5 weeks increased NMDAR/AMPAR ratio at the Schaffer collateral synapses in the hippocampus and facilitated the induction of LTP in the CA1 region [145]. The observed synergistic effect of both probiotics and prebiotics on improved learning and memory formation is also associated with an increase in butyrate production, which leads to increased BDNF levels and a decrease in hippocampal pro-inflammatory cytokines. Besides, SCFA production by gut bacteria following probiotic supplementation enhances LTP and modulates memory formation in male Sprague–Dawley rats and C57BL/6 mice, by increasing histone acetylation, which is a critically involved in memory formation [37]. Of note, depending on the microbiota context, SCFA may also exert deleterious effects. Thus, in GF APPPS1 mice, which display decreased Aβ-plaques and plasma concentration of SCFA in comparison to age-matched conventionally bred Specific Pathogen Free (SPF) APPPS1 mice, exogenous SCFA supplementation exacerbated Aβ-plaques deposition and microglia impairment. The latter manifested by decreased phagocytic capacity despite increased microglia accumulation in the vicinity of Aβ-plaques in SCFA-treated GF APPPS1 mice [146].

By analogy to probiotics, several bacterial metabolites or prebiotics have beneficial effects on cognitive functions in cognitively non-impaired animals or advanced stage of AD-like pathology in rodent models where the underlying mechanisms may involve the histone acetylation [147]. In addition, supplementing APP/PS1 mice with oligosaccharides from *Morinda officinalis* (OMO) for 6 months reverses the learning and memory deficits. OMO also regulates neurotransmitter secretion by influencing certain gut microbes that are implicated in the production and secretion of neurotransmitters such as monoaminergic neurotransmitters [148]. Interestingly, a four-months treatment with SLAB51 probiotic formulation (*Streptococcus thermophilus, Bifidobacteria*, *Lactobacilli*) during the pre-symptomatic stage increases fecal SCFA production (acetate, propionate and butyrate) in a mouse model of AD-like pathology (8 weeks old 3xTg-AD males) [149]. This probiotic formulation might therefore exert its action via an intermediate of SCFA prebiotic leading to amelioration of cognitive deficits in the novel object recognition test at 24 weeks, i.e. at the overt stage of pathology in this model [149]. Of note, an alternative explanation for the improved cognitive function upon SLAB51 treatment is the reduction of oxidative stress in the brain of treated AD-like mice [149]. Indeed, 8 weeks old 3xTg-AD mice supplemented for 16 weeks with SLAB51 showed an increase in Sirtuin-1 (SIRT1)-dependent mechanisms that promotes neuroprotective effects by lowering ROS production and promoting cell survival [150]. In a similar, more recent study using APP/PS1 model, 8 weeks administration of *Agathobaculum butyriciproducens* (SR79) significantly improved cognitive performance in the novel object recognition test and Y-maze at the overt stage of AD-like pathology, together with decreased Aβ plaque deposition and microglia activation. Of utmost importance, SR79 treatment significantly decreased the expression of IL-1β and complement component C1QB genes in the cortex of APP/PS1-treated mice [151].

Remarkably, probiotics may not only counteract certain AD-associated symptoms but they could also represent a promising therapeutic adjuvant in AD treatment. Thus, combining a probiotic strain (*Lactobacillus plantarum*) to FDA-approved AD drug memantine for 12 weeks reinforces its therapeutic benefits by attenuating cognitive deterioration and LTP deficits in APP/PS1 mice, in addition to improving impaired synaptic plasticity. This concomitant treatment modulates the gut microbiota composition and inhibits the synthesis of TMAO [152]. Moreover, associating *Lactobacillus plantarum* to memantine efficiently reduced intracerebral Aβ plaques and Aβ_42_/Aβ_40_ ratio. Neuroinflammation was further attenuated when the probiotic strain was used alone or in conjunction with memantine. These anti-inflammatory actions were associated with reduced plasma levels of clusterin [152], known to promote the accumulation of fibrillar Aβ aggregates in the brain of AD-like mice [153].

Some human studies have reported beneficial effects of probiotics on cognitive performance in AD patients although the effects were stage-dependent. Randomized, double blind and placebo-controlled trial in 60 AD patients (age range 60–95 years) used a probiotic preparation (*Lactobacillus casei, Lactobacillus acidophilus*, *Lactobacillus fermentum* and *Bifidobacterium bifidum*), which was administered (2 × 10^9^ CFU/g) to half of the subjects, the other half receiving placebo for 12 weeks. Probiotic-treated patients displayed statistically a significant improvement in cognitive MMSE score (+ 27.90% ± 8.07) in comparison to the placebo group whose score was slightly deteoriated (-5.03% ± 3.00). Interestingly, the probiotic treatment also decreased serum concentration of C-reactive protein (CRP), a general marker of systemic inflammation [154]. In contrast, in a more recent study using a similar, but enriched, probiotic preparation (*Lactobacillus casei, Lactobacillus acidophilus*, *Lactobacillus fermentum, Lactobacillus lactis, Lactobacillus paracasei, Lactobacillus plantarum, Lactobacillus salivarius, Bifidobacterium bifidum* and *2 strains of Bifidobacterium lactis*) and shorter (4 weeks) treatment in a cohort of 20 AD patients (age range 60–93 years), no significant alteration of intial MMSE score (18.5 ± 7.7) was reported [155]. Of note, the probiotic treatment yielded increase in *F. prausnitzii* in fecal specimens and altered serum biomarkers of immune activation. Serum kynurenine concentration decreased with concomitant increase in kynurenine / tryptophan ratio, which was further correlated with increased serum levels of the inflammatory marker, neopterin [155]. The authors suggested that probiotic treatment in this setting could be associated with macrophage and/or dendritic cell activation. These conclusions should nevertheless be taken with caution as the sample size was limited (*n* = 20 patients) and the study was not placebo-controlled [155]. In addition, when a similar (but not identical) probiotic preparation comprising *Lactobacillus acidophilus*, *Lactobacillus fermentum, Lactobacillus plantarum, Bifidobacterium bifidum, Bifidobacterium lactis* and *Bifidobacterium longum* was administred for 12 weeks to severe AD patients (*n* = 48) against placebo in a randomized, double-blind trial, there was no amelioration of the cognitive score (Test Your Memory, TYM scale) or change in serum level of pro- (TNFα, IL-6) or anti- (IL-10) inflammatory cytokines [156].

Of interest, when applied at the earlier stages of the pathology, a probiotic preparation of *Bifidobacterium breve* (strain A1) slowed cognitive decline in 19 (out of 27 enrolled) MCI subjects after 24-week supplementation [157]. Because this latter study was performed in an open-label, single-arm setting, these data have to be confirmed in a double-blind, randomized and placebo-controlled study before concluding on higher efficiency of such probiotic treatement if applied at early stages of AD.

However, it has to be stressed that in addition to the aforementioned beneficial or neutral effects, some studies have reported negative effects of probiotic treatment. In a double-blind, placebo-controlled trial with random allocation of subjects, probiotic preparation (*Lactobacillus casei*, Shirota strain: 6.5 × 10^9^ live bacteria) was given in a milk drink for 3 weeks to a cohort of healthy subjects (*n* = 124 at the end of the study) enrolled from general population (mean age 61.8 years ± 7.3; range 48–79 years). Two weeks after the end of the study, placebo group displayed significantly better cognitive performance in semantic memory test (Wechsler Memory Scale, 1998) than probiotic-treated group (p < 0.02) [158]. The reason for a negative impact of such a probiotic remain unclear, but the authors suggested it may be a “chance-effect” due to a relatively low number of subjects, thus calling for a need of study replication before drawing any definitive conclusions [158].

To sum-up, despite some promising results in human studies where cognitive performance was used as a read-out, as well as in animal models (in which the upstream mechanisms of cognitive functions could be assessed), there is currently no consensus on beneficial effects of probiotic treatments. This is likely inherent, at least in part, to the design of studies assessing such probiotics in different i) formulations; ii) concentrations; iii) treatment periods; iv) stages of AD pathology; v) read-outs, i.e. cognitive function or underlying cellular (LTP, LTD…) and molecular (synaptic receptors and proteins expression) processes. Future work is thus needed to ensure standardized probiotic formulations in pilot clinical studies which would allow for their comparison in order to draw conclusions on their impact, required in the light of their proposed clinical applications in AD [159].

**Fig. 3:**
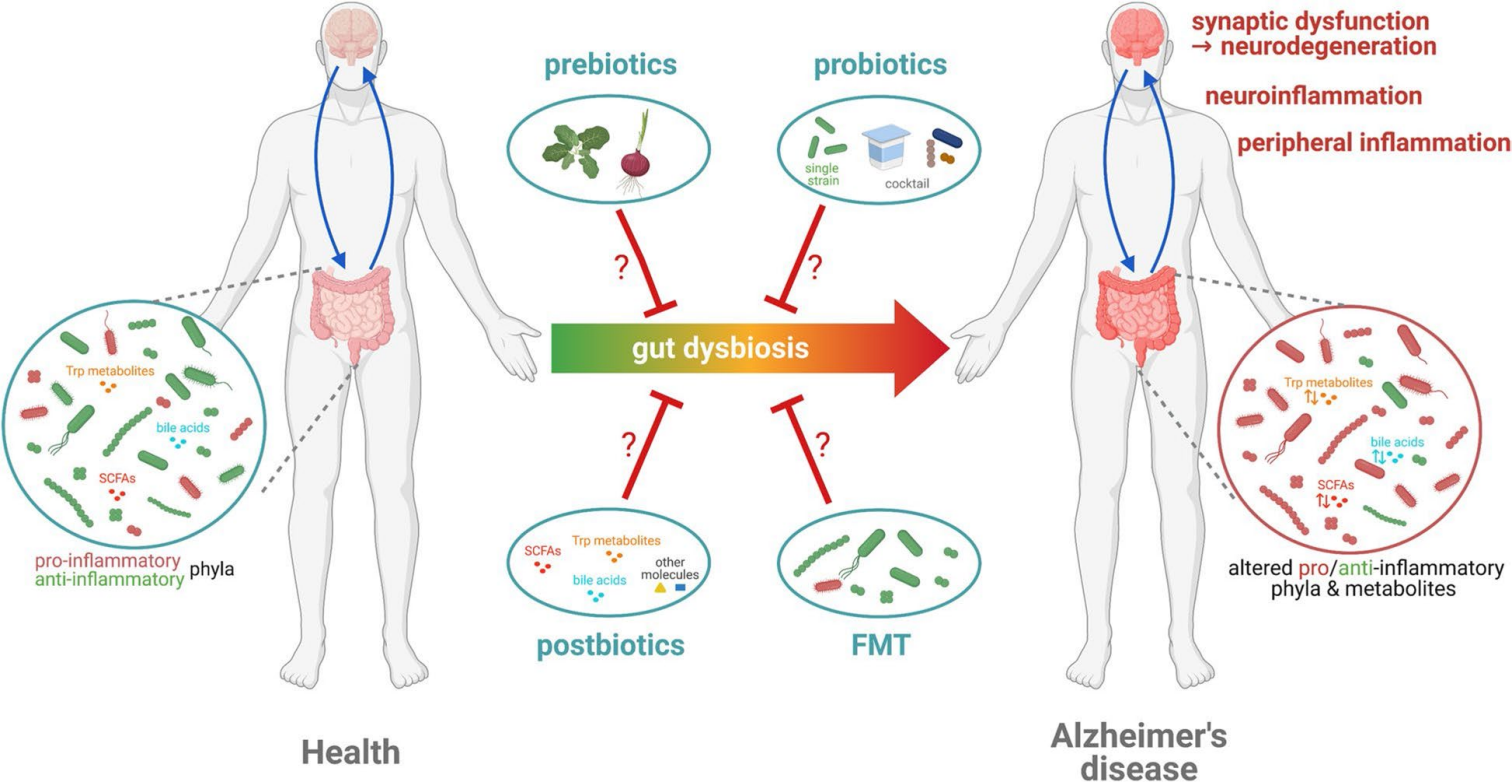
Therapeutic potential of putative microbiota-based interventions. AD is associated with gut microbiota dysbiosis characterised by increased pro-inflammatory (red microorganisms) and decreased anti-inflammatory (green microorganisms) phyla and altered microbial metabolites amounts. Although significant differences exist according to the geographical and ethnical factors, an increase in *Bacteroidetes* and decrease in *Firmicutes* (pro- and anti-inflammatory phyla, respectively) have been reported in AD patients by most studies (see AD and gut dysbiosis: link with neuroinflammation and involvement of microglia). Prebiotics (non-digestible fiber components of the food) have the capacity to stimulate the growth of microbiota with beneficial actions, such as, for example, SCFA-producing microorganisms. Probiotics are live microorganisms (single strain or multi-strain cocktails) providing beneficial effects to the host as for instance, *F. prausnitzii*. Postbiotics are metabolites produced by microbiota, such, as for example, SCFA. FMT consists of transferring fecal matter from a healthy donor to restore microbiota composition and function of patient. All these approaches could represent protective therapeutic strategies to prevent the shift towards detrimental peripheral inflammation, neuroinflammation, synaptic dysfunction and subsequent neurodegeneration and thereby, slow disease progression (for details, see Restoring AD-associated neuronal function by targeting microbiota?). Created with Biorender.com.

Moreover, it remains to be established whether beneficial effects of pro- and prebiotics could be further enhanced by healthy diets consisting in high intakes of plant-based foods, probiotics, antioxidants, soy beans, nuts, and omega-3 polyunsaturated fatty acids, combined with a low intake of saturated fats, animal-derived proteins, and refined sugars. Such diets are indeed known to decrease the risk of cognitive impairments and eventually the risk of AD [160] (Fig. 3).

#### FMT approach

There are currently only a few studies in which FMT was attempted to modify AD-like pathology in animal models. A recent study in APP/PS1 mice (aged 6 months) demonstrated a global beneficial impact on AD pathology, when FMT from age-matched healthy WT donors was administred over one-month after elimination of endogenous microbiota by a 3-day treatment with antibiotics [161]. Notably, FMT restored gut dysbiosis and fecal SCFA levels in treated APP/PS1 mice, alleviated cerebral accumulation of Aβ_40_ and Aβ_42_, tau-protein hyperphosphorylation, inflammatory markers (COX-2 and CD11b), and normalized decreased expression of pre- (synapsin-1) and post- (PSD95) synaptic proteins, as well as cognition [161]. Consistently, when the same APP/PS1 model was treated by FMT during pre-symptomatic stage (3 months of age) using donor fecal matter from 16 month-old APP/PS1 (corresponding to the overt pathology stage), a significant acceleration in accumulation of Aβ-plaques was seen [162]. Although there was no change in microglia, alteration of astrocyte morphology was observed, reminiscent of their functional impairment [162]. In agreement, when an opposite approach using 2 month-old WT mice as recipient and 9 month-old 5xFAD mice (i.e. overt stage of pathology) as fecal matter donor, FMT-treated WT mice displayed an impairment in cognitive function, which was associated with decreased hippocampal neurogenesis, increased microglia activation and pro-inflammatory (TNFα, IL-1β) cytokine expression both in the hippocampus and plasma [163]. The instrumental role of 5xFAD-derived microbiota in triggering cognitive dysfunction was further strengthened in this study by the absence of cognitive alterations in 2 month-old WT mice that received FMT from 9 month-old (healthy) WT mice [163]. Strikingly, quite different results were obtained in another experimental set-up, when antibiotic treatement was used not just to eliminate the endogenous microbiota and allow for succesfull engraftement of the transplantated microbiota to adult animals, but to achieve a long-lasting immunomodulation. In the latter paradigm, antibiotics were applied pre-weaning (2–3 post-natal week) and FMT administered one day after the end of antibiotic treatement to APPPS1 mice whereas the subsequent analyses were performed at at 9 weeks of age. The data obtained in antibiotic/FMT-treated APPPS1 mice indicated that Aβ amyloidosis and morphology of plaque-associated microglia and pre- (synaptophysin-positive) and post-synaptic (PSD95-positive) neurons were restored, independently of whether the donor of fecal matter was WT or APPPS1 mice [164]. The reasons for discrepancies between these studies [163, 164] remain unknown but are most likely related to the developmental stage (adult versus post-natal), different AD-like pathology models and antibiotic cocktail composition, individual antibiotic concentration and duration of treatement.

Considering human studies, FMT received a single approved indication so far—i.e. the treatment of recurrent *Clostridioides difficile* infection. More recently, the research has focused on other therapeutic avenues including in AD, although no such FMT studies are published so far in human. The unique previous study (NCT03998423; https://www.clinicaltrials.gov/) had unfortunately to be interrupted due to severe acute respiratory syndrome coronavirus 2 (SARS-CoV2) pandemia. Neverheless, FMT studies in animal models of AD-like pathology showed globally (although there are yet some discrepancies between the studies, see above) encouraging results in terms of reducing neuroinflammation, Aβ accumulation and synaptic dysfunction [143, 161–165].

Overall, the available data point to manipulating microbiota and/or their metabolites as a potential innovative, attractive and non-invasive therapeutic approach for restoring optimal synaptic plasticity and associated cognitive functions in AD, at least indirectly by modulating innate and adaptive immunity both at the periphery and in the CNS (**Fig. ****3**. However, most available data from animal studies relate only to advanced pathological stages and future research is needed to assess the early stages of pathogenesis. Moreover, similar to approaches using pro- and prebiotics, standardization is also urgently needed in the field of FMT. The parameters that have to be standardized include: i) stage of AD pathology eligible for FMT; ii) preparing the gut environment of the recipient (bowel cleansing *versus* antibiotics, composition, concentration, duration) to optimize donor microbiota engraftement; iii) origin of fecal matter (from single or multiple donors, fresh vs frozen,….); and iv) genotype of donors (e.g. presence or exclusion of genetic risk factors). The present lack of standardization may indeed seriously hamper or slow down progress in the field.

## Conclusions and future directions

Currently available treatments for AD are mostly symptomatic and offer relatively limited benefits. It is thus essential to develop innovative and earlier applicable therapies, with a hope that beginning such treatments before the onset of major cognitive impairments could improve their efficacy. As alterations in the gut microbiota can induce changes in brain activity, manipulating the gut microbiome has arisen as a potential therapeutic target in AD. Encouraging data using different strategies to modulate microbiota (pro- or prebiotic treatments, FMT, etc.…) appear useful in ameliorating cognitive deficits even when applied at advanced stages of AD-like pathology in rodent models. Recently reported findings support that microbiota alterations are detectable during the pre-symptomatic stage in such animal models [106–108], strongly suggesting that gut microbiota modification during the earliest stages of AD pathogenesis may also be beneficial. Early modification of microbiota during the latent pre-symptomatic stage may therefore become a promising therapeutic approach for the treatment of AD.

In addition, a possible use of vagus nerve stimulation as a mean of direct modulation of microbiota-brain interaction for therapeutic purposes has recently emerged. The role of the vagus nerve in the direct communication between brain and gut is now widely recognized [4, 7]. Auricular stimulation of the vagus nerve is a non-invasive approach that is increasingly used in therapeutic handling of specific neurological and neuropsychiatric conditions [166]. This therapy relies on the anatomy of the vagus nerve that has a common cranial trunk with a sensory cervical branch from the ear [167–169]. Remarkably, the vagus nerve stimulation has been already applied for 6–12 months in cohorts of AD patients and yielded significant cognitive-enhancing effects [170, 171]. Future studies are still needed to decipher the underlying mechanisms, and notably whether they may include microbiota changes.

In conclusion, both indirect and direct communications between intestinal microbiota and the CNS along the gut-brain axis (Fig. 2) provide a rationale for non-invasive and affordable therapeutic innovations in CNS disorders. These could be exploited more broadly by public health policies not only in AD, but also in the context of other neurological and psychiatric disorders in which microbiota dysbiosis has been reported. Although such dysbiosis, manifesting as an imbalance between pro- and anti-inflammatory phyla, may implicate different taxa in different neurodegenerative diseases, the outcomes of neurodegeneration-related dysbiosis appear similar. They involve a shift towards pro-inflammatory state in the gut, yielding increased gut permeability and subsequent triggering of peripheral inflammatory response, which in turn impacts neuroinflammation and impair neuronal function. In this light, targeting microbiota to achieve immunomodulation indirectly, rather than targeting directly immune functions, appears as an attractive possibility as microbiota is upstream of peripheral inflammation / neuroinflammation. Inducing direct immunomodulation without turning down the upstream pro-inflammatory dysbiosis may indeed be less effective than targeting microbiota to counteract its pro-inflammatory components. However, this should not exclude combinatorial immuno-modulatory approaches that could target both microbiota and the immune system, which may turn out to be more efficient than a single-targeted approach.

## Data Availability

Not applicable.

## Abbreviations

Aβ: Amyloid-beta
AD: Alzheimer’s disease
AMPA: α-Amino-3-hydroxy-5-methyl-4-isoxazolepropionic acid
AMPARs: AMPA receptors
APCs: Antigen-presenting cells
ApoE: Apolipoprotein E
BBB: Blood–brain-barrier
BMAA: β-N-methylamino-L-alanine
CCL5: CC-motif chemokine Ligand-5
CCL11: CC-motif chemokine Ligand-11
CNS: Central Nervous System
CSF: Cerebro-spinal fluid
CXC5: CXC-motif chemokine Ligand-5
DAM: Disease-Associated Microglia
DAMPs: Damage-associated molecular patterns
EPSP: Excitatory post-synaptic potential
FMT: Fecal microbiota transplantation
FXR: Farnesoid-X-receptor
GABA: Gamma-aminobutyric acid
GABARs: GABA receptors
GH: Germ-free
GPBAR1: G protein-coupled bile acid receptor 1
GWAS: Genome-wide association studies
IFNγ: Interferon gamma
IgA: Immunoglobulin A
IL-1α: Interleukin-1 alpha
IL-1β: Interleukin-1 beta
IL-1Ra: IL-1 receptor antagonist
IL-2: Interleukin-2
IL-3: Interleukin-3
IL-4: Interleukin-4
IL-5: Interleukin-5
IL-6: Interleukin-6
IL-10: Interleukin-10
IL-12: Interleukin-12
IL-13: Interleukin-13
IL-17: Interleukin-17
IL-18: Interleukin-18
LPS: Lipopolysaccharide
LTD: Long-term depression
LTP: Long-term potentiation
MAMPs: Microbe-associated molecular patterns
MCI: Mild Cognitive Impairment
MCP-1: Monocyte chemoattractant protein-1
MCT: Monocarboxylate
MGnD: MicroGlia in neuroDegeneration
MHC: Major histocompatibility complex
MIP-1α: Macrophage Inflammatory Prtein 1-alpha
MMSE: Mini-mental state examination
NALP3: NACHT, LRR and PYD domains containing protein (now renamed as NLPR3: NLR family pyrin domain containing 3)
NFT: Neurofibrillary tangles
NMDA: N-methyl-D-aspartate
NMDARs: NMDA receptors
NOS: Nitrogen reactive species
OMO: Oligosaccharides from *Morinda officinalis*
PAMPs: Pathogen-associated molecular patterns
PRRs: Pattern Recognition Receptors
RNA: Ribonucleic acid
ROS: Reactive oxygen species
SARS-CoV2: Severe acute respiratory syndrome coronavirus 2
SCD: Subjective Cognitive Decline
SCF: Stem Cell Factor
SCFA: Short Chain Fatty Acids
SCMT: Sodium-coupled monocarboxylate transporters
SIRT1: Sirtuin-1
SPF: Specific Pathogen Free
SRs: Scavenger receptors
TGFβ: Transforming growth factor beta
Th1: T-helper 1
Th2: T-helper 2
Th17: T-helper 17
TLR2: Toll-like receptor-2
TLR4: Toll-like receptor-4
TMAO: Trimethylamine N-oxide
TNFα: Tumour necrosis factor alpha
TNFR1: TNFα type-1 death receptor
Tregs: Regulatory T-cells
TREM2: Triggering receptor expressed on myeloid cells 2
YKL-40: Chitinase-3-like protein 1
